# A Physiological System of Nosology; with a Corrected and Simplified Nomenclature

**Published:** 1817-10

**Authors:** 


### PART II.

### COMPREHENSIVE ANALYTICAL REVIEW
OF
### MEDICAL LITERATURE.

"Tros, tyriusve, nobis nullo discrimine agetur."

*A Physiological System of Nosology ; with a corrected and simplified Nomenclature.* By JOHN MASON GOOD, F.R S. Mem. Am. Phil. Societ. and F. L. S. Philadelphia. One Vol. Octavo. pp. 584. London, 1817.

WHEN we cast a retrospective glance at even a small portion of the stream of time that has rolled towards us, and have observed——

System on System into ruin hurl'd,

As regularly and as certainly, as century succeeds century, or progeny progenitors ; we find it difficult to account for that secret principle which prompts us to expend so much labour, learning, health, wealth, and irrevocable time, in the construction of literary edifices, which, however majestic or beautiful in themselves, have their foundations necessarily washed by an ever-moving tide that must shortly overwhelm them in the ocean of oblivion. Yet, as this melancholy reflection applies to every sublunary pursuit, and characterizes mankind in every age, and in every country, it is evident that the impulse is from the hand of Nature—nay, from the hand of God ;—and consequently productive of GOOD, upon the whole.

Oh, blindness to the future—*wisely* given !

If Providence has permitted the ambition of a Buonaparte to deluge Europe with blood ; and the genius of a Brown to poison medicine with hypothesis, will any person affirm, that no moral benefit has resulted to the political world from the one, or that no rays of light have been shed on the nebulous points of medical science by the other ?

But whatever may be the objections to medical *Theories,* on account of the pernicious influence which they occasionally exert on medical practice, there cannot, we conceive, be any reasonable cavil raised against the *Nosological arrangement* of diseases. We would compare Nosology to the scaffolding which surrounds the edifice of a medical education, *during its construction.* There, it is essentially necessary, and singularly useful. But *after* the building is complete, and comes to be *practically* inhabited, we fear the nosological scaffold is as useless in the metaphorical, as it would be undecorative in the architectural sense of the word. As it too often happens, that men kick away, with ingratitude, the ladder by which they have ascended to fame or eminence; so some modern physicians, forgetful of the advantages of nosology in the *study* of diseases, have proscribed it as altogether a useless artifice, both in *that*, and in the treatment of them.

But whatever degree of relative importance may be attached to the *Nosological part* of the work before us, Mr. Good has taken care to store it with such a mass of excellent matter, in the shape of *notes*, that were a pen run through the whole of the former, the latter alone would stamp a value on the book, which could not fail to prove a passport to every medical library in the kingdom. The learning, the research, the discrimination, portrayed throughout every page of these interesting notes, must raise the author to a very high, and a very honourable seat in the temple of medical literature.

In the present Article, we shall confine ourselves to an analysis of Mr. Good's "preliminary discourse," which is a most masterly composition. We hope in this miniature representation, to catch so much of the spirit, sense, and even expression of the original, as to enable the reader to judge for himself, and induce to a contemplation of the full length picture.

*Prelim. Discourse.* The main object of the following work is not so much to interfere with existing systems of nosology, as to fill up a vacant niche in the gallery of physiological study, by connecting the science of diseases more closely with the sister branches of natural knowledge; by giving it a more assimilated character, intelligible classification, simple, yet comprehensive arrangement, and more correct nomenclature.

SECT. I. *Nosological Systems.* The simplest system, if worthy of that name, is the *alphabetic*—instance the important work of Dr. Heberden. Another modification has been founded on *the duration of diseases*, as divided into acute and chronic—instance the works of Aretæus, and Cælius Aurelianus. A third modification is grounded on *anatomy*, as has been done by Sennert and Morgagni. A fourth, on the *causes* of disease, as in the schools of Boerhaave, Hoffman, and Riverius. Latterly, the *distinctive symptoms* of diseases have been adopted as the basis of nosology—instance Sauvages, Linnæus, Cullen, &c. This last basis is the only one that will generally hold true to itself. Of the seat of diseases we often know but little—of their causes less; but there are certain marks or characters in their usual progress, which uniformly accompany and distinguish them, to which the epithet *pathognomonic* has been correctly applied.

II. Plater may be considered as the morning star that first glimmered on the hemisphere of syptomatology. His *Praxis Medica*, though an imperfect sketch of symptomatic nosology, was published in 1602. Sydenham, if he did not avail himself of it, was actuated by the same quickening spirit, since his epistles and treatises are practical comments upon Plater's principle.

III. Sauvages's *Nosologia Methodica* followed in 1731, but was only completed in 1768. This last edition is indeed an Herculean labour. It is arranged on three plans; symptomatical, etiological, and anatomical, to meet the taste of the old and new schools. The symptomatical, which is extensively elucidated, comprises ten classes; upwards of forty orders; more than three hundred genera, and an almost innumerable host of species—"*quel nombre prodigieux d'ennemis!*" as M. De Ratte exclaimed!

Diffuseness is the leading error of this system; yet it is a venial one, for this amplitude rendered it, when first completed, a sort of nosological Bazaar, to which every one might have recourse for what article he wanted. Notwithstanding its imperfections, the student who neglects to read it, neglects one of the most important branches of his education. Passing over the amendments which were attempted by Selle, Halle, Macbride, &c. &c. we come to Cullen.

IV. Cullen determined on changing and recasting the system of Sauvages from its commencement. He reduced the classes from ten to four; and, influenced throughout the whole of his reform by the spirit of simplicity and concentration, he narrowed the forty-four orders of Sauvages to twenty, and his three hundred and fifteen genera to one hundred and fifty-one. He next carried his pruning hook into the field of species, where the reduction bore an equal proportion to that in the genera. The new system was now started in the largest medical school of Europe, its author presiding at the head of it. It instantly rushed into popularity, and became a subject of general approbation. "Its aim at simplicity, as well in extent as in arrangement, was noble, and bespoke correct views, and a comprehensive mind." The author shewed that he had laboured his attempt in no ordinary degree; many of his definitions discovered a mastery that had never before been exemplified; pictures painted to the life, and of proper dimensions.

Yet the system has faults, and insurmountable ones. The two first classes, indeed, have considerable merit; yet Dr. C. has frequently found himself under the necessity of over-stepping the natural outlines or *arrondissements,* and invading adjoining districts; a palpable instance of which occurs in the very class with which he commences:

"For the tribe of HÆMORRHAGES, which forms one of its orders, has no direct catenation with any idea suggested by PYREXIA, in the common use of the term; they require coercion to bring them into a state of union; and what is still worse, Dr. C. with all the force he could employ, has found himself incapable of coercing more than one half of them; and, consequently, has been obliged to leave the other half behind, or rather to banish them for contumacy, to the extreme region of his fourth class." xv.

In the 3d Class, the term CACHEXIÆ has been, and still continues to be, used in senses so extremely different by different writers, that it by no means suggests to the mind a connected group of diseases. But the most faulty part of Dr. Cullen's arrangement is in the class LOCALES. The sole object of this class is to form an Appendix for the purpose of receiving such genera as the foregoing classes could not include. Indeed he even acknowledges, that there are diseases "*sufficiently well known, for which a fit place cannot be found in his system.*" This is a defect from which no time or labour can ever relieve it. He has, moreover, given entrance to diseases that have very little claim to admission; the consequence is, a sad medley, and, in many cases, not the least affinity or family resemblance; instance *psora* and *fractura,* which follow in immediate succession. "Dr. Cullen, however, has found that they are both *local* disorders, which entitle them to a common class."

It is impossible to take a survey of Dr. Cullen's system without noticing his very extraordinary confusion of genera and species; and Dr. C. is not the only nosologist of the present day who has run into this error. Dr. C. has given genera without species. Now, he who describes a genus that has no species belonging to it, or gives a generic without a specific name, describes a mere abstract form, a thing that has no existence without the addition of other signs or qualities which do not enter into his definition. For a genus is not a disease, any more than it is an animal, a vegetable, or a mineral; but a group or assemblage of any of these, possessing certain like characters, and associated in consequence of such resemblance.

"Of a want of systematic precision the examples are very frequent in every class. Thus synocha, synochus, hectica, phrenitis, hepatitis, and many more, afford instances in the first class; hypochondriasis, chlorosis, and others in the second; polysarcia, hydrometra, hydrocele, &c. in the third; pica, adipsia, profusio, and nearly all that follow, in the fourth class. Nor can it be said, that in these cases we are tacitly referred to the species of Sauvages for adoption; since under *adipsia* we are cautioned against using the only one in Sauvages that has any relation to Cullen's genus; and in *profusio* we have a genus for which Sauvages has no direct parallel. It would be easy to prove, that a very considerable number of these naked genera are in reality species belonging, or which a very little dexterity might make to belong, to another genus." p. xxii.

It is unnecessary to offer more examples. We need not wonder why the distinguished reputation of Dr. Cullen should be incapable of securing to his nosological system the popularity with which it was at first erected, nor why a host of learned rivals, few of whom have humiliated him by their competition, in different parts of Europe, have endeavoured to offer schemes big with the fair promise of realizing the noble object he had in view, and free from the defects he has exhibited. None of these rival attempts have been eminently successful; while the greater part have dropped from the cradle into the grave.

After passing over the systems of Selle, Plouquet, and Pinel, the second of which "is singularly distinguished by the author's fondness for long crabbed words," we come to those of our own countrymen, Macbride, Crichton, and Darwin. The author of Zoonomia, says Mr. Good, was a man of great genius, daring imagination, and extensive reading; but unfortunately stung with a desire of distinguishing himself by seeing things, weighing things, and combining things, in a manner different from every one else. In short, he would, at any time, rather think wrong with himself, than think right with other people. Hence, while he offers much to gratify, he offers also much to offend; and proves, that if he had aimed at less he would have accomplished more.

"The entire basis is theoretical; in several parts visionary: The whole *may*, therefore, prove hereafter to be unfounded; a considerably portion of it evidently *is* unfounded at present. But the direct death-warrant of the system consists in his making every single proximate effect (in common language proximate cause, or symptom) a distinct disease; for as the same proximate effect or symptom may be produced by several, or by each of what Darwin calls proximate causes, and which constitute his classes, it follows, that the very same species or specific disease must in such cases belong equally to some order or other of several, or of all the classes of his system. And such, to the student's embarrassment and surprise, he will find upon examination to be the real fact. Thus, while *variola* (small-pox) is arranged under cl. ij. ord. j. gen. iij. *eruptio variolæ* (small pox eruption) occurs under class iv. ord. j. gen. ij. So *hydrophobia* appears first in j. iij. j. and afterwards iij. j. j. *Diabetes* in j. iij. ij. and again in iv. iij. j. *Palpitation of the heart* in j. ij. j. and again in j. iij. iij. being twice in the same class; and so of many others.

"Such perplexity sets all the ordinary laws of method at defiance; yet it is easily accounted for from the nature of the primary divisions. It is not, however, so easy to account for Dr. Darwin's introducing into his table of diseases such natural states or affections as the following: Healing of ulcers, j. j. iij. Deglutition, ij. j. j. Respiration under the same genus. And the life of an egg, iv. j. iv. which, from some unaccountable fancy, is thus rendered one of the DISEASES OF ASSOCIATION. While, to make the system still more defective and incapable of practical use, its author has given us neither his specific nor his generic definitions, excepting, indeed, occasionally; confining himself entirely to his Latin and English names; and sending us for their *descriptions* to "The Nosologia Methodica of Sauvages, and the Synopis Nosologiæ of Dr. Cullen, and the authors to which they refer." p. xxx.

This is a hard sentence on the illustrious Darwin! And yet we are among those who think, that the *greater number* of Darwin's illustrations of disease, his rationes symptomatum; and above all, his methodi medendi, will "not moulder, like the structures already erected, into the sand of which they were composed, but stand unimpaired, a rock amid the waste of ages."

We must pass over Mr. Good's remarks on the nosological arrangements proposed by Parr and Young; and also on the limited systems of Plenck, Willan, Abernethy, Bateman, &c. Nor can we do justice to his very able and critical sections on *medical nomenclature*, by attempting their analysis. We must therefore refer to the volume itself, which we trust will soon be in the hands of most medical readers. We may just say by the way, however, that in his reformation of nomenclature, he has been guided—1st. by a strict adherence to Greek and Latin terms alone. 2d. A use of as few technical terms as possible, and consequently a forbearance from all synonyms. 3d. A simplification of terms, as far as it can be done without violence or affectation, both in their radical structure and composition. 4th. An individuality and precision of sense in their respective use.

*Arrangement of Diseases.* A knowledge of the animal frame involves a comprehensive acquaintance with anatomy, physiology, and nosology or pathology. The first teaches us the structure, the second the functions, and the third the diseases of the corporeal fabric. To accomplish an incorporation of the three different studies is the object of the following system; the whole edifice of which will be erected on a physiological basis. The primary divisions of this system are intended to take a physiological range, and follow up the diseases of the animal fabric in the order in which the physiologist usually developes its organization and functions. But from what point are we to set out? Shall we pursue, with Haller, the growing ens through all its rising stages of evolution and elaboration to its maturity of figure and sensation, or take, at once, the animal frame in its mature and perfect state, and trace it from some well-defined and prominent function, through all the rest; which, like links in a circular chain, may be said to issue from it, and to be dependent on its existence and properties?—The latter has been chosen by our author.

To repair the common wear and tear of life, a due proportion of food must be supplied to the alimentary canal. Its elaboration here is termed the DIGESTIVE FUNCTION. The diseases of this function will constitute the *first* class in the following System.

As the chyle resulting from the foregoing elaboration has yet to be operated upon by the atmospheric air, the FUNCTION OF RESPIRATION furnishes the diseases of the *second* class.

The blood, now matured, is returned to the heart, and sent forth, in a circular course, to every organ of the body, as the common pabulum from which it is to secern what it stands in need of; the waste blood being carried back to the fountain from which it issued. It is this circulatory track that constitutes the SANGUINEOUS FUNCTION; and its diseases form the *third* class of the ensuing pages.

But the blood does not circulate by its own power. From the brain, which it recruits and refreshes, its vessels (perhaps itself) receive a perpetual influx of that sensorial energy which gives motion, as the blood gives food, to the entire machine; converts the organized into an animal and intellectual system, and forms the important sphere of the NERVOUS FUNCTION. This function affords scope for a large family of diseases, and hence a ground-work for our *fourth* class.

We now arrive at the SEXUAL FUNCTION; and obtain from the diseases by which it is marked, a *fifth* class.

As it is necessary that the antagonist processes of restoration and detrition should maintain a fair balance, the minute secretory and absorbent vessels hold the same relation to each other as the arteries and veins, and conjointly create an EXCERNENT FUNCTION, whose diseases lay a foundation for the *sixth* class.

It yet remains to create a class for EXTERNAL ACCIDENTS, and those ACCIDENTAL MISFORMATIONS, which occasionally disfigure the fœtus in the womb. This constitutes the *seventh* class; and under these seven classes it will possibly be found that all the long list of diseases may be included which man is called to suffer, or the art of medicine to counteract.

"The succession is here easy and natural: every class, at least with the exception of the last, leads immediately to that which follows it; and the student will at once comprehend its scope, and readily retain its arrangement. The order is strictly physiological." lx.

Some of the genera are new, for the purpose of enlisting under so many common banners, diseases which have hitherto been held as distinct genera, but which ought rather to have been regarded as species, as adypsia and polydipsia. Bulimia, anorexia, pica, cardialgia, &c. The aim of the author, in short, has been "to generalize as far as the thread of connexion would allow; but never to congregate where its continuity is broken off." It must be remembered that the principle of this arrangement consists in determining the proper class of a disease from the *general function* that is injured, and not from the *parti-*

*cular organ*, which only regulates the subordinate divisions; and that, where two general functions are injured at the same time, that constitutes the class which appears to be most prominently affected. Thus strophulus and scabies, which seldom extend deeper than the secretory vessels of the skin, belong necessarily to the class ECCRITICA, or that comprising diseases of the excernent function; while variola and rubeola, though equally occupying the surface, belong to the class HÆMATICA, or the diseases of the sanguineous function; which, in both these cases, is primarily and chiefly affected, as is obvious from the pyrectic action of the heart and arteries.

As it was an object with the author to diminish rather than to multiply genera, as far as consistent with perspicuity, the family of DOLORES or LOCAL PAINS is entirely suppressed, and its genera and species distributed under other heads, as mere symptomatic affections.

In order to render the work still more useful, Mr. Good has subjoined to the systematic name of every disease its chief technical and vernacular synonyms. This is a new attempt, and we think a highly praise worthy one. The vernacular synonyms are confined to English, German, and French. The technical are derived from Greek, Latin, and Arabic tongue, the three most extensive languages of antiquity. The Arabic is given in its own as well as in Roman characters. The Arabic is the oldest living language in the world, and was certainly studied and cultivated as the medium of polite science, and particularly of medical learning, at the splendid court of Solomon. It descended as a living language through the dark ages; and ou the resurrection of science, towards the 15th century, it was from Arabic sources and the Saracenic schools that Europe again became acquainted with the medical treasures of the Grecian writers. It is easy, therefore, to trace the path by which the Arabic and other Oriental terms have found their way into the medical and botanical vocabularies of the present day.

But there is another, and a very prominent feature in the present work, which we have already glanced at—namely, the Notes.

"In order to afford relief to the dryness of technical definitions, and verbal criticisms, the author has digested his notes into a running commentary, which he has endeavoured to render replete with interesting cases, valuable hints or remarks, and singular physiological facts, gleaned from a pretty extensive perusal of the most approved authorities, collective or individual, ancient or modern; occasionally interwoven with similar illustrations, as they have occurred to the writer in his own private walk and intercourse of life." *Prelim. Discourse. p. 97.*

The following passage is deserving of attention.

"There has, of late years, been an unnecessary degree of scepticism thrown over several of the public Journals, or Ephemerides, to which the author has occasionally referred, in consequence of their containing much of the *marvellous*, and reposing on the sole authority of the individual by whom the case or article is related. It should be remembered, however, that several of these collectanea were established for the express purpose of communicating *singular and extraordinary facts or occurrences* in natural history or physiology, and of confining themselves to such cases. It is true, that they are for the most part published upon the responsibility of the writer alone; but it is upon the same single credit that by far the greater number of cases are given which are published in our own day, whether by individuals, or by collective bodies. Even the Royal Society, from whom the ensuing pages have, perhaps, chiefly borrowed, does not, in communicating its Transactions to the public, assume a higher degree of testimony; and thinks it prudent, by a special advertisement prefixed to every half volume, to caution its readers that its Committee do not pretend 'to answer for the certainty of the facts, or propriety of the reasonings contained in the several papers, which must still rest on the credit or judgment of their respective authors.' This, indeed, ever has been, and ever must be, the main ground of authority and rational assent; and on this account, while the writer has selected from a somewhat extensive range, he has endeavoured, as far as he has been able, to weigh the characters and stations of the different individuals from whom he has quoted, whether as isolated reporters, or members of scientific academies: though it is highly probable, after all, that the greater number of the most curious and extraordinary aberrations from the common laws of health and disease, introduced into the ensuing commentary, will be found drawn from well-established scientific miscellanies and transactions of our own day, rather than from those of earlier periods." p. xcviij.

We have thus presented a well-defined outline of Mr. Good's preliminary discourse, omitting, however, the greater part of his learned and critical disquisitions, for which we must refer to the work itself. From this sample, many of our readers will be induced to anticipate our next month's labour, in exhibiting some faint outline of the Nosological part, by perusing the original; and indeed we can confidently predict, that few medical libraries will refuse this volume a place, and that few Medical

Readers, of any literary taste or zeal, will fail to peruse, and often afterwards refer to, the ingenious, the learned, and the useful work of Mr. John Mason Good.

